# Pharmacological modulation of p75 neurotrophin receptor in microglial cells improves resilience to rotenone cytotoxicity

**DOI:** 10.3389/fphar.2026.1895046

**Published:** 2026-07-20

**Authors:** Alessio Valenza, Daniele Pensabene, Francesca Rendina, Maurizio Muzzi, Anna Fracassi, Sandra Moreno, Marco Segatto

**Affiliations:** 1 Department of Science, University Roma Tre, Rome, Italy; 2 LIFE Institute for Research and Health Care - Santa Lucia IRCCS, Rome, Italy; 3 Department of Biosciences and Territory, University of Molise, Pesche, Italy; 4 Mitchell Center for Neurodegenerative Diseases, Department of Neurology, University of Texas Medical Branch, UTMB, Galveston, TX, United States; 5 Moody Brain Health Institute, UTMB, Galveston, TX, United States; 6 Graduate School of Biomedical Sciences, UTMB, Galveston, TX, United States

**Keywords:** LM11A-31, microglia, neuroinflammation, oxidative stress, p75NTR, Parkinson’s disease, rotenone

## Abstract

**Introduction:**

Parkinson’s disease (PD) is a progressive neurodegenerative disorder, characterized by dopaminergic neuronal loss, mitochondrial dysfunction, oxidative stress, and chronic neuroinflammation. Microglial activation plays a primary role in disease progression, by amplifying inflammatory response and redox imbalance. The low-affinity neurotrophin receptor p75NTR has recently emerged as a potential therapeutic target in neurodegenerative disorders, due to its involvement in cell survival, apoptosis, and inflammatory signaling. In the present study, we investigated whether pharmacological modulation of p75NTR by LM11A-31 could protect microglial cells against Rotenone (Rot)-induced toxicity, a widely used tool to mimic PD.

**Methods:**

BV2 microglial cells were exposed to 50 nM Rot in the presence or absence of 0.5 μM LM11A-31. Cell viability, apoptotic signaling, oxidative stress, inflammatory response, and cytoskeletal organization were evaluated using immunofluorescence, Western blotting, TUNEL assay, and Scanning Electron Microscopy (SEM).

**Results:**

Rot exposure significantly increased p75NTR expression and induced marked microglial dysfunction, characterized by activation of apoptosis, oxidative stress, and inflammatory phenotype. LM11A-31 treatment improved cell survival and reduced apoptotic features, as shown by decreased TUNEL^+^ cells and cleaved caspase-3 immunoreactivity. In parallel, LM11A-31 restored microglial morphology and cytoskeletal integrity, also reducing ultrastructural alterations induced by Rot. Moreover, p75NTR modulation significantly blunted microglial activation markers, including Iba1 and CD68. LM11A-31 also reduced oxidative stress by limiting NOX-related signaling and lipid peroxidation, while partially restoring antioxidant defenses through modulation of Nrf2, PPARα, and glutathione-associated pathways.

**Conclusion:**

Our findings demonstrate that pharmacological modulation of p75NTR by LM11A-31 protects microglial cells against Rot-induced cytotoxicity and inflammatory activation. These protective effects involve the rescue of redox balance, suppression of pro-inflammatory signaling, preservation of cytoarchitecture, also resulting in increased cell survival. Overall, targeting p75NTR may represent a promising therapeutic strategy to counteract microglial dysfunction and neuroinflammatory processes associated with Parkinson’s disease.

## 1 Introduction

Parkinson’s disease (PD) is a chronic, progressive neurological disorder of the central nervous system (CNS) and the second most prevalent neurodegenerative condition worldwide (Poewe et al., 2017). First described in 1817 by James Parkinson, PD has traditionally been defined by motor symptoms, including bradykinesia, resting tremors, and postural instability, but is now recognized as a multisystem disorder, including cognitive, autonomic, and neuropsychiatric symptoms (Ben-Shlomo et al., 2024). Despite the clinical and etiological heterogeneity of PD, common pathological mechanisms are recognized. Hallmark features include the progressive degeneration of dopaminergic neurons in the substantia nigra pars compacta and the intracellular accumulation of α-synuclein aggregates in the form of Lewy bodies (Geibl et al., 2024; Bengoa-Vergniory et al., 2017).

PD arises from a complex interplay between genetic susceptibility and environmental factors. Approximately 5%–10% of cases are monogenic and driven by mutations in genes such as *SNCA, LRRK2, VPS35, PRKN, PINK1*, and *PARK7*. Studies of these mutations have provided key insights into core pathogenic mechanisms, including energy dysmetabolism, protein misfolding, altered autophagy, and cellular stress responses (Jia et al., 2022; Morris et al., 2024). At the same time, environmental insults, particularly exposure to neurotoxic compounds such as pesticides, have been implicated in PD risk and progression, as supported by epidemiological and experimental evidence (Dorsey et al., 2023; Ibarra-Gutiérrez et al., 2023; Paul et al., 2023). Among these molecules, Rotenone (Rot) has emerged as a prototypical environmental toxin linked to PD. Thanks to its ability to cross the blood–brain barrier, Rot inhibits mitochondrial respiration by targeting complex I of the electron transport chain (ETC.). This leads to decreased ATP production, altered calcium homeostasis in dopaminergic neurons, and overproduction of reactive oxygen species (ROS). Its direct mitochondrial toxicity, as well as the consequent robust neuroinflammatory response mediated by glial cells, ultimately leads to dopaminergic neuron loss (Maturana et al., 2015). Such features make Rot a widely used experimental tool for modeling PD and designing therapeutic strategies targeting the disturbed molecular pathways underlying the disease (Johnson and Bobrovskaya, 2015).

Mitochondrial dysfunction is a central event in PD pathogenesis. Impaired mitochondrial respiration increases electron leakage, thereby promoting ROS generation and progressive disruption of redox homeostasis (Subramaniam and Chesselet, 2013; Alqahtani et al., 2023; Henrich et al., 2023). When ROS production exceeds the capacity of antioxidant defense systems, oxidative damage builds up, affecting lipids, proteins, and nucleic acids (Trist et al., 2019). Post-mortem analyses of PD brains consistently reveal elevated oxidative modification markers and decreased ATP levels, supporting oxidative stress as a sustained and convergent pathogenic mechanism in PD (Sofic et al., 2006).

Oxidative stress and mitochondrial dysfunction, together with the misfolded α-synuclein accumulation, are associated with neuroinflammatory responses, and may interact bidirectionally with immune activation in the CNS (Liang et al., 2026). In this context, the role of glial cells in modulating inflammatory processes is essential to resist the insult and mantain tissue functionality. Particularly, under basal conditions, microglia reside in a resting state (M0), characterized by a highly ramified morphology and the continuous extension and retraction of their processes to monitor the CNS microenvironment (Pallarés-Moratalla and Bergers, 2024). In response to harmful stimuli, microglia become activated and undergo pronounced morphological and functional remodeling (Wang et al., 2023). This activation reflects their dual role in CNS pathology: microglia may contribute to tissue protection and repair through the clearance of damaged cells, supporting tissue remodeling, and restoration of homeostasis, while also potentially exacerbating injury through the release of pro-inflammatory mediators (DiSabato et al., 2016; Gao et al., 2023). Although this functional dichotomy has traditionally been described according to the M1 pro-inflammatory and M2 anti-inflammatory/reparative classification, increasing evidence indicates that microglial phenotypes *in vivo* are highly heterogeneous, dynamic, and strongly dependent on the specific pathological context (Stratoulias et al., 2019; Xu et al., 2023). In PD and other neurodegenerative diseases, persistent microglial activation has been shown to reinforce mitochondrial dysfunction and oxidative stress, leading to a vicious cycle that accelerates neurodegenerative progression (Peterson and Flood, 2012).

Besides the traditional inflammatory pathways, increasing evidence supports the involvement of neurotrophins in the molecular mechanisms underlying PD pathophysiology. Perturbation in neurotrophins signaling can significantly influence the progression of PD-associated phenotypes (Numakawa and Kajihara, 2025). Neurotrophins exert their biological actions by binding to two main classes of membrane receptors: the high-affinity tropomyosin receptor kinase (Trk) family and the low-affinity p75 neurotrophin receptor (p75NTR). Even though the involvement of high-affinity receptors in PD has been extensively investigated (Ali et al., 2024a), the role of p75NTR in PD-associated oxidative stress and neuroinflammation is yet to be fully explored. p75NTR is a shared receptor for all neurotrophins and, in addition to its well-established functions in regulating neuronal apoptosis, differentiation, and growth (Ali et al., 2024b), emerging studies suggest its participation in pathways associated with oxidative stress and antioxidant response in PD models (Pensabene et al., 2025).

Due to its emerging role in neurodegenerative processes, p75NTR has been proposed as a potential pharmacological target, given its action toward distinct intracellular signaling cascades. Several small molecules have been reported to modulate p75NTR-dependent pathways linked to neuronal damage while promoting pro-survival signaling (Longo and Massa, 2013). Among these compounds, LM11A-31, designed to interact with the NGF loop 1 binding domain, represents the most extensively investigated p75NTR modulator to date. LM11A-31 selectively engages NF-κB- and PI3K-dependent signaling pathways while sparing Trk-mediated pathways (Massa et al., 2006).

Although LM11A-31 has demonstrated robust therapeutic efficacy in experimental models of Huntington’s and Alzheimer’s diseases (Knowles et al., 2013; Simmons et al., 2025), its role in PD has been explored only in dopaminergic neurons (Pensabene et al., 2025), and no studies are yet available in the context of microglial perturbation. In the present study, we investigated the potential neuroprotective effects of LM11A-31 in a Rot-induced microglial cell model of PD. We demonstrate that pharmacological modulation of p75NTR by LM11A-31 attenuates Rot-driven oxidative stress and suppresses inflammation associated with mitochondrial dysfunction, counteracting pro-degenerative signaling pathways, and promoting the restoration of microglial homeostasis.

## 2 Materials and methods

### 2.1 Cell cultures

The murine BV2 microglial cell line was cultured at 37 °C in a humidified atmosphere with 5% CO_2_ in high-glucose DMEM (D6429, Merck Life Science) supplemented with 10% (v/v) heat-inactivated fetal bovine serum (FBS; F7524, Merck Life Science) and 1% (v/v) penicillin/streptomycin (P06-07100, PAN Biotech). For all experiments, cells were grown to 80%–90% confluency and subjected to no more than 20 cell passages.

Three experimental conditions were set: Control (Ctrl), Rotenone-treatment (Rot), and Rotenone + LM11A-31 treatment (Rot + LM11A-31). Cells were seeded and maintained in DMEM supplemented with 2% FBS. After 24 h, Ctrl and Rot cultures received vehicle alone (DMSO, final dilution 1:1000), whereas Rot + LM11A-31 cultures were pre-treated with 0.5 µM LM11A-31 (SML0664, Sigma-Aldrich). At 48 h, the medium was refreshed and Ctrl cultures continued to receive vehicle alone, Rot cultures were treated with 50 nM Rot (R8875, Merck Life Science), and Rot + LM11A-31 cultures received a co-treatment of Rot (50 nM) together with LM11A-31 (0.5 µM). All cells were collected at 72 h.

### 2.2 Cell count

BV2 cells were seeded in six-well plates, and morphological changes were monitored using a Primovert phase-contrast inverted microscope (Carl Zeiss, Germany) at ×20 magnification. Representative images were captured for each experimental condition. For cell counting, cultures were incubated with trypsin for 5 min and the resulting cell suspensions were collected and mixed. Cell numbers were determined using a Bürker chamber, performing three independent counts per experimental group, and the mean value was used for quantitative analysis.

### 2.3 Immunofluorescence

For immunofluorescence analyses, 150,000 cells were seeded onto sterilized poly-L-lysine–coated coverslips (P6282, Merck Life Science). After treatments, cells were fixed with 4% paraformaldehyde (D1408, Merck Life Science) in PBS for 5–10 min at room temperature (RT), prior to permeabilization with 0.1% Triton X-100 (X100, Merck Life Science) in phosphate-buffered saline (PBS; D8537, Merck Life Science) for 5 min. To minimize non-specific antibody binding, samples were incubated for 1 h at RT in a blocking solution of 3% bovine serum albumin (A3912, Merck Life Science) and 0.1% Triton X-100 in PBS.

Cells were then incubated overnight at 4 °C with primary antibodies in blocking solution, as listed in Table 1. After PBS washes, coverslips were incubated for 1 h at RT with appropriate fluorophore-conjugated secondary antibodies (goat anti-mouse Alexa Fluor 555, A28180, Thermo Fisher Scientific; goat anti-rabbit Alexa Fluor 488, A27034, Thermo Fisher Scientific). Nuclei were counterstained with DAPI (D9542, Merck Life Science) or Hoechst (H3570, Invitrogen) and coverslips were mounted using Fluoroshield Mounting Medium (F6182, Merck Life Science). Fluorescence images were acquired using a confocal microscope (Eclipse Ti2, Nikon) equipped with a ×40 objective. Image capturing was performed with NIS-Elements software (Nikon) on a Windows 10 platform. The same imaging parameters were consistently maintained across experimental groups. Fluorescence intensity was quantified using ImageJ (version 1.54 days, National Institutes of Health). Data were expressed as the ratio of mean fluorescence intensity to cell area and normalized to Ctrl values.

**TABLE 1:** List of antibodies employed in this work.

Antibody	Dilution	N° catalog	Company	Technique
BDNF	1:100	ab108319	Abcam	IF
Catalase	1:10,000	200401051	Rockland immunochemicals	WB
CD68	1:100	ab283654	Abcam	IF
Caspase-3	1:100	C8487	Sigma-aldrich	IF
GAPDH	1:10,000	ab8245	Abcam	WB
GPx-1	1:100	ab22604	Abcam	IF
GSH	1:150	ab19534	Abcam	IF
GSR	1:50	sc-133245	Santa cruz biotechnology	IF
GSS	1:50	sc-166882	Santa cruz biotechnology	IF
IBA1	1:50	sc-32725	Santa cruz biotechnology	IF
IL-6	1:50	sc-32296	Santa cruz biotechnology	IF
NOX2	1:1000	sc-130543	Santa cruz biotechnology	WB
NOX4	1:50	sc-518092	Santa cruz biotechnology	IF
NRF2	1:100	sc-365949	Santa cruz biotechnology	IF
P22^PHOX^	1:50	sc-130551	Santa cruz biotechnology	IF
p47^PHOX^	1:50	sc-17844	Santa cruz biotechnology	IF
p75NTR	1:100	sc-271708	Santa cruz biotechnology	IF
PPAR-α	1:100	PA1-822A	Invitrogen	IF
PPAR-β/δ	1:100	ab23673	Abcam	IF
PPAR-γ	1:100	sc-7273	Santa cruz biotechnology	IF
SOD1	1:200	ab13498	Abcam	IF
SOD2	1:1000	ab13533	Abcam	WB
TNF-α	1:100	sc-52746	Santa cruz biotechnology	IF
TrkB	1:50	sc-12	Santa cruz biotechnology	IF
TrxR1	1:300	sc-28321	Santa cruz biotechnology	WB
α-tubulin	1:500	T5168	Sigma-aldrich	IF

### 2.4 Cell lysate and western blot assay

Cell lysis and Western blot were based on previously described protocols (Pallottini et al., 2020; Colardo et al., 2023; Colardo et al.,2025). BV2 cells were lysed by sonication for 30 s in CelLytic buffer (C2978, Sigma-Aldrich), supplemented with protease and phosphatase inhibitor cocktails, to obtain total protein extracts. Protein concentration was determined using the Lowry assay. Samples were then mixed with Laemmli buffer and denatured by boiling at 95 °C for 5 min. Equal amounts of protein (20 µg) were separated by 10% SDS–PAGE and transferred onto nitrocellulose membranes using the Trans-Blot Turbo system (Bio-Rad Laboratories). Membranes were then incubated overnight at 4 °C with primary antibodies, diluted in blocking solution, as listed in Table 1. After washing with PBS-T, membranes were incubated for 1 h at RT with horseradish peroxidase (HRP)-conjugated secondary antibodies (anti-mouse, 1706516, Bio-Rad Laboratories; anti-rabbit, 1706515, Bio-Rad Laboratories). Immunoreactive bands were detected using Clarity ECL Western blotting Substrate (1705061, Bio-Rad Laboratories). Images were acquired, and densitometric analyses were performed using ImageJ (version 1.54 days, National Institutes of Health). Densitometric values were expressed in arbitrary units (a.u.) as the ratio of target protein band intensity to GAPDH and normalized to Ctrl values.

### 2.5 Scanning electron microscopy (SEM)

For SEM analysis, cells were plated on coverslips and processed according to our previously described protocol (Colasuonno et al., 2020). Briefly, cells were fixed with 2.5% glutaraldehyde in 0.1 M cacodylate buffer, pH 7.4, for 45 min at 4 °C. After washes, samples were post-fixed with 1% OsO_4_ in the same buffer for 45min at 4 °C in the dark. Cells were then dehydrated through a graded ethanol series, followed by immersion in hexamethyldisilazane. After air-drying, slides were mounted on aluminum stubs using double-sided conductive adhesive carbon discs and sputter-coated with gold using an Emitech K550 Sputter Coater. Electron micrographs were acquired with a Helios 5 CX FIB-SEM (Thermo Fisher Scientific) using secondary-electron detection at 5 kV.

### 2.6 TUNEL assay

Detection of apoptotic cells by TUNEL assay (G3250, Promega) was performed as previously reported (Marioli et al., 2024). Following experimental treatments, BV2 cells were fixed with 4% formaldehyde in PBS for 10 min at RT, then permeabilized with 0.2% Triton X-100 in PBS for 10 min at 4 °C. After PBS washes, slides were immersed in Equilibration Buffer for 5 min at RT and then incubated with a reaction mixture containing 45 µL Equilibration Buffer, 5 µL Nucleotide Mix, and 1 µL terminal deoxynucleotidyl transferase (TdT), for 1 h at 37 °C. The reaction was blocked by incubation with 2× saline–sodium citrate (SSC) buffer for 15 min. Finally, nuclei were counterstained with Hoechst (1:10,000 dilution) for 10 min at RT prior to mounting and imaging.

### 2.7 BODIPY staining

BODIPY 581/591 C11 (D3861, Thermo Fisher Scientific) was dissolved in DMSO to obtain a 2 mM stock solution and diluted in culture medium to a final concentration of 5 µM. Cells were incubated with the staining solution for 40 min at 37 °C, washed with PBS, and nuclei were counterstained with Hoechst. Fluorescence images were acquired using a confocal microscope, and raw images were analyzed with ImageJ to obtain quantitative data based on the green/red fluorescence intensity ratio. Ratio values were normalized to the corresponding control samples.

### 2.8 MitoSOX™ red staining

Mitochondrial superoxide production was assessed using the MitoSOX™ Red mitochondrial superoxide indicator (M36008, Thermo Fisher Scientific, United States), following a previously described protocol (He et al., 2021).

### 2.9 Phalloidin staining

BV2 cells were seeded on poly-L-lysine–coated coverslips. After treatment, cells were fixed and incubated with Acti-stain™ 488 Fluorescent Phalloidin (PHDG1, Cytoskeleton) to label actin filaments, according to the manufacturer’s instructions.

### 2.10 Statistical analysis

Data are presented as mean ± standard deviation (SD). All analyses were performed on a minimum of 3 biological replicates (unless otherwise specified), each one corresponding to an independent cell culture experiment performed in different days. Normality of data was assessed using the Shapiro-Wilk test. Comparisons between two experimental groups were performed using an unpaired two-tailed Student’s T-test, while comparisons among the three experimental groups were performed using one-way analysis of variance (ANOVA), followed by Tukey’s *post hoc* test. Exact N values are reported in figure legends. Statistical analyses were performed using GraphPad Prism version 8.4.2 (GraphPad Software) for Windows 10 and a p-value <0.05 was considered statistically significant.

## 3 Results

### 3.1 p75NTR modulation ameliorates apoptotic morpho-functional features after rot exposure in BV2 cell line

**FIGURE 1:**
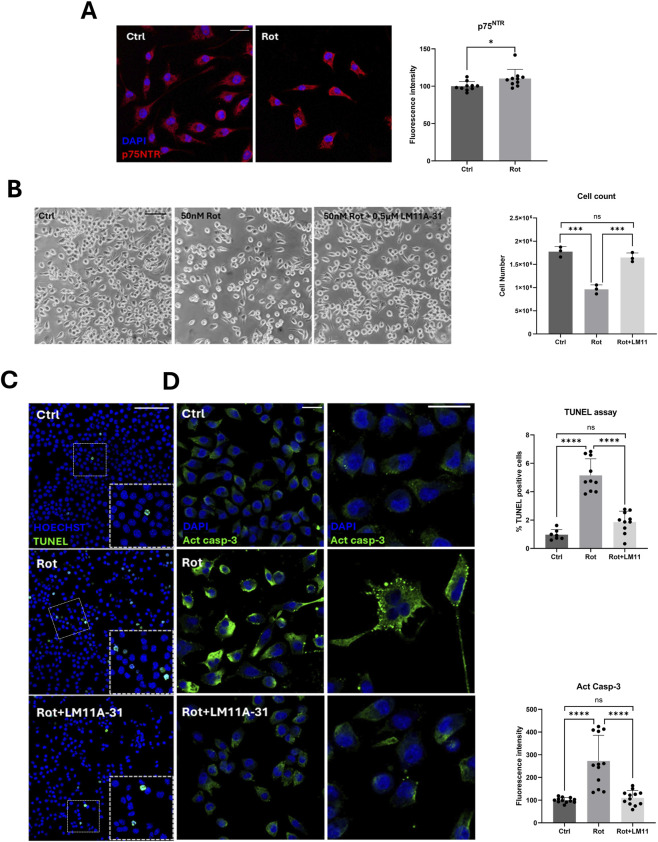
Anti-apoptotic effect of LM11A-31 in BV2 microglial cells treated with Rot. **(A)** Representative confocal microscopy images (left panel) and quantitative analysis (right panel) of p75NTR expression (red) in BV2 cells treated with vehicle (Ctrl, DMSO) or 50 nM rotenone (Rot) for 24 h. N = 10. Magnification: ×40. Scale bar = 25 μm. Statistical analysis was performed using an unpaired Student’s t-test. **(B)** Representative brightfield images (left panel) and cell count quantification (right panel) of BV2 cells treated with vehicle (Ctrl, DMSO), 50 nM rotenone (Rot), or co-treated with LM11A-31 (Rot+LM11A-31, 0.5 μM) for 24 h. N = 3. Magnification: ×20. Scale bar = 50 μm. **(C)** Representative TUNEL assay images (green) of BV2 cells treated as described above. Hoechst (blue) was used for nuclear counterstaining. N = 7–10. A minimum of 4000 cells was counted. Magnification: ×20. Scale bar = 100 μm. **(D)** Representative confocal microscopy images of active caspase-3 staining in BV2 cells treated as previously described, left panel shows a low-magnification representative field, whereas the right panel shows a higher-magnification view from a different microscopic field. DAPI (blue) was used to counterstain nuclei. N = 12. Magnification: ×40. Scale bar = 25 μm. Data are presented as mean ± SD. Dots represent individual biological replicates. Statistical analysis was performed using one-way ANOVA followed by Tukey’s *post hoc* test. *p < 0.05; ***p < 0.001; ****p < 0.0001.

We first characterized our Rot-treated BV2 model by assessing p75NTR expression through immunofluorescence analysis. Elevated p75NTR signal (Figure 1A) was detected following Rot exposure, compared to control conditions, indicating receptor induction by neurotoxic stress.

To evaluate whether pharmacological targeting of p75NTR could mitigate Rot toxicity, we first established a suitable working concentration of the small-molecule ligand LM11A-31 in BV2 cells. Based on cell number analysis, LM11A-31 exerted a significant protective effect against Rot-induced toxicity at 0.5 μM and 1 μM, whereas lower concentrations were ineffective (Supplementary Figures S1, S2). Based on these experiments, 0.5 μM was selected for all subsequent experiments as the lowest effective concentration capable of significantly counteracting Rot insult. Consistently, total cell number (Figure 1B) decreased significantly following Rot treatment, while co-administration of LM11A-31 restored cell numbers to values comparable to those of untreated cells, supporting a protective effect based on p75NTR modulation. Apart from p75NTR, other neurotrophin signaling pathways, namely, TrkB/BDNF, are scarcely affected by the treatments, since the receptor levels are unchanged, while a slight increase in BDNF is detected (Supplementary Figure S3). Noteworthy, LM11A-31 treatment failed to significantly alter either TrkB receptor or BDNF levels, compared to Rot alone.

Given the established role of p75NTR in pro-apoptotic signaling pathways, we subsequently evaluated whether the observed decrease in cell number was associated with apoptotic cell death. TUNEL assay (Figure 1C) showed a marked increase in DNA fragmentation in cells treated with Rot, whereas LM11A-31 largely reduced the number of TUNEL-positive nuclei, thereby restoring values close to those of the control group. Consistently, immunofluorescence for cleaved caspase-3 (Figure 1D) showed increased signal in cells treated with Rot, displaying a small vesicle-like appearance, especially localized near the plasma membrane. Co-treatment with LM11A-31 significantly reduced such immunoreactivity, corroborating the anti-apoptotic effects highlighted by TUNEL assay findings.

**FIGURE 2:**
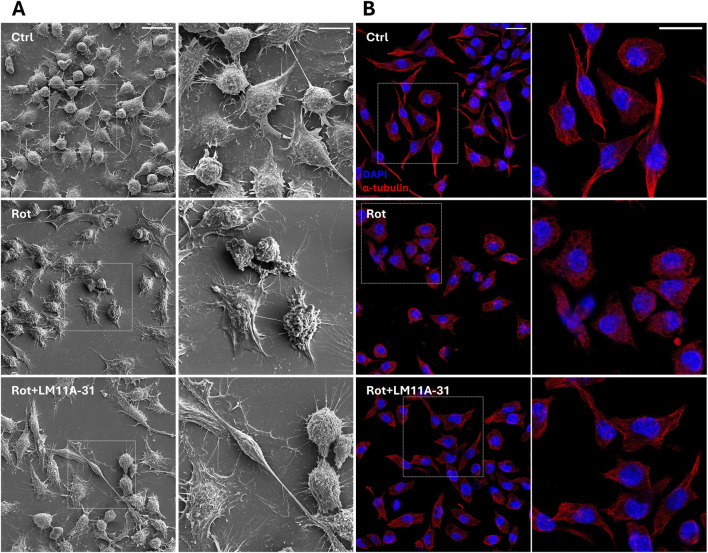
LM11A-31 rescues Rot-induced morphological alterations, while restoring cytoskeletal architecture. **(A)** Representative scanning electron microscopy (SEM) images of BV2 cells treated with vehicle (Ctrl), 50 nM rotenone (Rot), or co-treated with LM11A-31 (Rot+LM11A-31) for 24 h. Left panels: lower magnification; right panels: higher magnification. Scale bars = 50 μm (left panels) and 25 μm (right panels). **(B)** Representative confocal microscopy images of α-tubulin staining (red) in BV2 cells treated as previously described. DAPI (blue) was used for nuclei counterstaining. Magnification: ×40. Scale bars = 25 μm.

To gain a deeper understanding of Rot-related morphological alterations, we conducted an ultrastructural analysis by scanning electron microscopy (SEM) to characterize potential alterations in cell surface (Figure 2A). Control cells showed an elongated shape with a relatively smooth surface numerous lamellipodia and filopodia, as well as extensive cell–cell contacts. In contrast, Rot treated cells exhibited reduced intercellular contacts, an irregular surface, and a rounded morphology accompanied by fewer and shorter membrane projections compared to Ctrl. Notably, Rot exposure induced classical apoptotic features, including membrane blebbing and apoptotic bodies. Co-treatment with LM11A-31 markedly improved cellular ultrastructural features, showing the restoration of an elongated cell shape, reducing surface vesiculation, increasing cell processes, and reducing the occurrence of apoptotic bodies.

To visualize the microtubule network, essential for maintaining cellular integrity, we also performed α-tubulin immunofluorescence (Figure 2B). While control cells showed a well-structured microtubule network with a filamentous, organized distribution across the cytoplasm, the tubulin network appeared disorganized in Rot-treated cells. Consistent with SEM observations, LM11A-31 co-administration resulted in the recovery of a more organized, continuous microtubule network, with α-tubulin immunodistribution comparable to Ctrl conditions.

### 3.2 Rot-induced microglial reactive phenotype is counteracted by LM11A-31 administration

**FIGURE 3:**
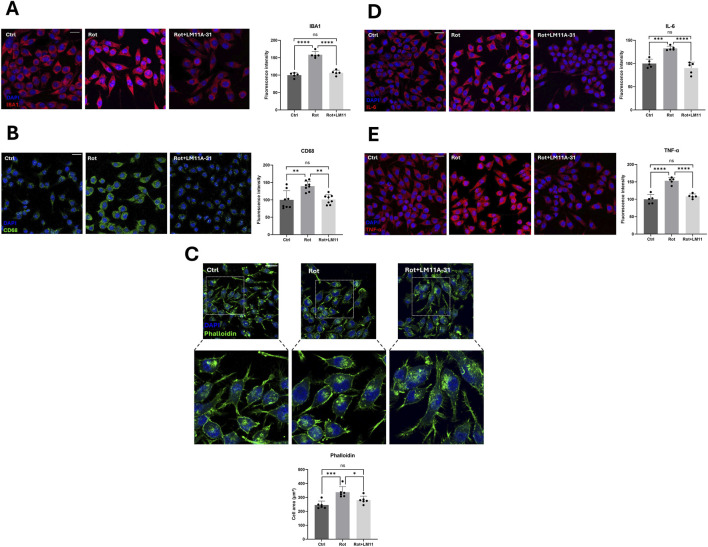
LM11A-31 influences the BV2 microglial phenotype. **(A)** Representative confocal microscopy images (left panel) and quantitative fluorescence intensity analysis (right panel) of IBA1 staining (red) in BV2 cells treated with vehicle (Ctrl), 50 nM rotenone (Rot), or co-treated with LM11A-31 (Rot+LM11A-31) for 24 h. DAPI (blue) was used for nuclear counterstaining. N = 5. Magnification: ×40. Scale bar = 25 μm. **(B)** Representative confocal microscopy images (left panel) and quantitative fluorescence intensity analysis (right panel) of CD68 staining (green) in BV2 cells treated as described above. DAPI (blue) was used to counterstain nuclei. N = 8. Magnification: ×40. Scale bar = 25 μm. **(C)** Representative confocal microscopy images of Phalloidin staining (green) showing actin cytoskeleton organization in BV2 cells following the indicated treatments. DAPI (blue) was used for nuclear staining. Higher magnification images are shown in the lower panels. Quantification of cell area is reported in the graph below. N = 6. Magnification: ×40. Scale bar = 25 μm. **(D)** Representative confocal microscopy images (left panel) and quantitative fluorescence intensity analysis (right panel) of IL-6 staining (red) in BV2 cells treated as previously described. DAPI (blue) was used for nuclear counterstaining. N = 5. Magnification: ×40. Scale bar = 25 μm. **(E)** Representative confocal microscopy images (left panel) and quantitative fluorescence intensity analysis (right panel) of TNF-α staining (red) in BV2 cells following the indicated treatments. DAPI (blue) was used for nuclear staining. N = 5. Magnification: ×40. Scale bar = 25 μm. Data are presented as mean ± SD. Dots represent individual biological replicates. Statistical analysis was performed using one-way ANOVA followed by Tukey’s *post hoc* test. *p < 0.05; **p < 0.01; ***p < 0.001; ****p < 0.0001.

We examined Rot-triggered microglial activation in BV2 cells by immunofluorescence staining for Iba1 and CD68, well-established markers of reactive microglia (Figures 3A,B). Fluorescence intensity for both proteins remarkably increased in Rot-treated cells compared to Ctrl. These observations were reinforced by phalloidin staining for F-actin visualization (Figure 3C). While Ctrl cells displayed a regular and well-organized actin cytoskeletal network, Rot administration induced alterations and remodeling of actin, including the appearance of O-ring-like structures and the loss of cellular projections, both evocative of a cytoskeletal remodeling associated with microglial activation. In contrast, co-exposure to LM11A-31 recovered a regular F-actin organization and also promoted an increased presence of lamellipodia and filopodia compared to Rot treatment. Importantly, morphometric analysis based on phalloidin staining allowed to quantitatively assess cellular areas showing a significant enlargement of cell area in Rot-exposed cells, which was effectively mitigated by p75NTR modulator, restoring cell size to Ctrl values.

To get further insights into the inflammatory response, we investigated the immunoreactivity levels for tumor necrosis factor-α (TNF-α) and interleukin-6 (IL-6) expression (Figures 3D,E). Immunofluorescence analysis showed a significant upregulation of both cytokines in Rot treated cells compared to controls. Consistent with the effects observed for the above-mentioned activation markers, LM11A-31 abrogated the enhanced signals of TNF-α and IL-6, restoring levels comparable to the Ctrl condition.

### 3.3 LM11A-31 blunts oxidative stress in BV2 microglial cells following rot treatment

**FIGURE 4:**
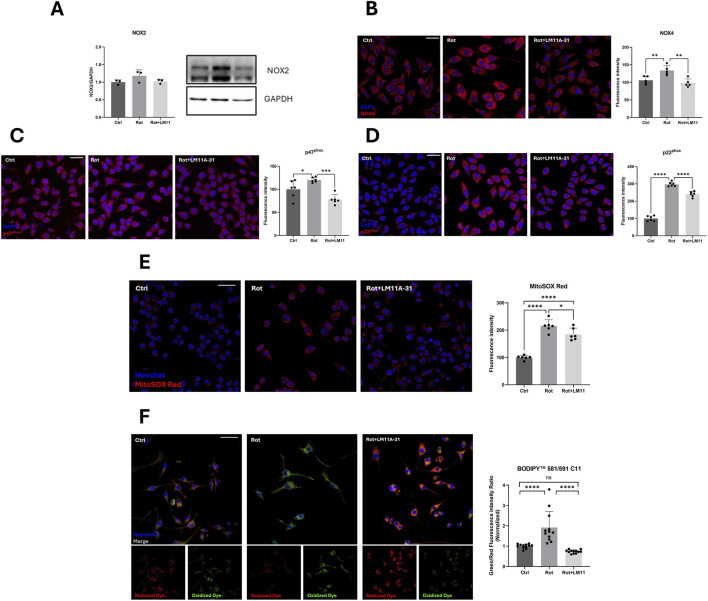
Rot-dependent increase in oxidative stress markers is suppressed by LM11A-31. **(A)** Representative Western blot analysis and densitometric quantification of NOX2 protein expression in BV2 cells treated with vehicle (Ctrl), 50 nM rotenone (Rot), or co-treated with LM11A-31 (Rot+LM11A-31) for 24 h. GAPDH was used as loading control. N = 3. **(B)** Representative confocal microscopy images (left panel) and quantitative fluorescence intensity analysis (right panel) of NOX4 staining (red) in BV2 cells treated as described above. DAPI (blue) was used for nuclear counterstaining. N = 5. Magnification: ×40. Scale bar = 25 μm. **(C)** Representative confocal microscopy images (left panel) and quantitative fluorescence intensity analysis (right panel) of p47^PHOX^ staining (red) in BV2 cells following the indicated treatments. DAPI (blue) was used for nuclear staining. N = 6. Magnification: ×40. Scale bar = 25 μm. **(D)** Representative confocal microscopy images (left panel) and quantitative fluorescence intensity analysis (right panel) of p22^PHOX^ staining (red) in BV2 cells treated as previously described. DAPI (blue) was used for nuclear counterstaining. N = 6. Magnification: ×40. Scale bar = 25 μm. **(E)** Representative confocal microscopy images and quantitative fluorescence intensity analysis of MitoSOX Red staining, used to detect mitochondrial superoxide production, in BV2 cells. Hoechst (blue) was used for nuclear counterstaining. N = 6. Magnification: ×40. Scale bar = 50 μm. **(F)** Representative confocal micrograph of BODIPY™ 581/591 C11 staining in BV2 cells treated with vehicle (Ctrl), rotenone (Rot), or co-treated with LM11A-31 (LM11A-31) for 24 h. Hoechst (blue) was used for nuclear staining. Representative images of reduced dye (red) and oxidized dye (green) are shown in the lower panels. Quantification of the green/red fluorescence intensity ratio is reported in the graph on the right. N = 12. Magnification: ×40. Scale bar = 50 μm. Data are presented as mean ± SD. Dots represent individual biological replicates. Statistical analysis was performed using one-way ANOVA followed by Tukey’s *post hoc* test. *p < 0.05; **p < 0.01; ***p < 0.001; ****p < 0.0001.

Oxidative stress primarily arises from excessive ROS production. As NADPH oxidases (NOXs) are a major source of ROS, we addressed possible activation of these enzymatic complexes by Rot. We assessed the expression levels of NOX2 and NOX4 (Figures 4A,B) and their respective regulatory subunits, p47^PHOX^ and p22^PHOX^ (Figures 4C,D). Western blot (WB) analysis showed only a non-significant increasing trend in NOX2 protein levels in Rot treated BV2 cells compared with controls, while immunofluorescence analyses showed a significantly enhanced signal for p47^PHOX^. Similarly, both NOX4 and p22^PHOX^ immunoreactivity levels were significantly increased following Rot exposure. Notably, LM11A-31 treatment effectively counteracted these changes, significantly reducing the levels of p47^PHOX^, NOX4, and p22^PHOX^, bringing them close to control values.

MitoSOX™ Red staining was then performed to specifically evaluate mitochondrial superoxide production (Figure 4E). Rot exposure induced a marked increase in MitoSOX™ Red fluorescence intensity compared with control cells, indicating enhanced ROS generation. Co-treatment with LM11A-31 significantly reduced the Rot-induced MitoSOX™ Red signal, although fluorescence levels remained higher than controls, suggesting a partial attenuation of mitochondrial superoxide accumulation. To further assess whether ROS production led to oxidative damage, lipid peroxidation was evaluated using the BODIPY™ 581/591 C11 probe (Figure 4F). Rot treatment markedly increased the green/red fluorescence ratio, indicative of enhanced lipid oxidation and oxidative stress. Conversely, LM11A-31 significantly mitigated BODIPY oxidation, restoring fluorescence values close to control levels, thereby supporting its protective effect against oxidative damage.

**FIGURE 5:**
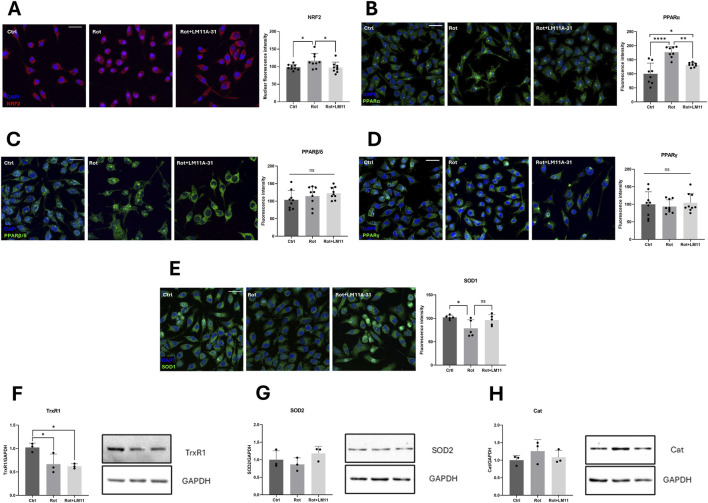
LM11A-31 partially restores antioxidant defense responses in BV2 microglial cells. **(A)** Representative confocal microscopy images (left panel) and quantitative fluorescence intensity analysis (right panel) of NRF2 staining (red) in BV2 cells treated with vehicle (Ctrl), 50 nM rotenone (Rot), or co-treated with LM11A-31 (Rot+LM11A-31) for 24 h. DAPI (blue) was used for nuclear counterstaining. N = 9. Magnification: ×40. Scale bar = 25 μm. **(B)** Representative confocal microscopy images (left panel) and quantitative fluorescence intensity analysis (right panel) of PPARα staining (green) in BV2 cells. DAPI (blue) was used for nuclear counterstaining. N = 8. Magnification: ×40. Scale bar = 25 μm. **(C)** Representative confocal microscopy images (left panel) and quantitative fluorescence intensity analysis (right panel) of PPARβ/δ staining (green) in BV2 cell line. DAPI (blue) was used for nuclear staining. N = 9. Magnification: ×40. Scale bar = 25 μm. **(D)** Representative confocal microscopy images (left panel) and quantitative fluorescence intensity analysis (right panel) of PPARγ staining (green) in BV2 cells. DAPI (blue) was used for nuclear counterstaining. N = 9. Magnification: ×40. Scale bar = 25 μm. **(E)** Representative confocal microscopy images (left panel) and quantitative fluorescence intensity analysis (right panel) of SOD1 staining (green) in BV2 cells. DAPI (blue) was used for nuclear staining. N = 5. Magnification: ×40. Scale bar = 25 μm. **(F)** Representative Western blot analysis and densitometric quantification of TrxR1 protein expression in BV2 cells treated as previously described. GAPDH was used as loading control. N = 3. **(G)** Representative Western blot analysis and densitometric quantification of SOD2 protein expression in BV2 cells following the indicated treatments. N = 3. **(H)** Representative Western blot analysis and densitometric quantification of catalase (Cat) protein expression in BV2 cells treated as previously described. N = 3. Data are presented as mean ± SD. Dots represent individual biological replicates. Statistical analysis was performed using one-way ANOVA followed by Tukey’s *post hoc* test. *p < 0.05; **p < 0.01; ****p < 0.0001.

Based on the observed induction of oxidative stress by Rot, we investigated whether LM11A-31 could modulate endogenous antioxidant defenses (Figure 5). Since oxidative stress reflects an imbalance between pro-oxidant and antioxidant systems, we evaluated key transcription factors responsible for managing cellular redox homeostasis and neuroprotective responses.

We first examined nuclear factor erythroid 2–related factor 2 (Nrf2), a major redox sensor and mitochondrial quality controller. Immunofluorescence showed that Rot exposure increased Nrf2 expression, whereas LM11A-31 treatment restored Nrf2 levels toward basal conditions, counteracting the alterations induced by Rot (Figure 5A). We then assessed the expression of members of the peroxisome proliferator-activated receptor (PPAR) family, which regulate antioxidant and anti-inflammatory pathways. LM11A-31 mitigated the Rot-induced upregulation of PPARα, while promoting its partial cytosolic redistribution (Figure 5B). Conversely, neither Rot nor LM11A-31 treatments significantly altered the overall expression levels of PPARβ/δ or PPARγ (Figures 5C,D).

To assess whether the observed modifications in antioxidant transcription factors were associated with alterations in downstream effector enzymes, we next examined key proteins involved in redox balance. Rot exposure significantly decreased SOD1 levels (Figure 5E), an essential enzyme for cytosolic superoxide detoxification. Notably, LM11A-31 treatment reversed these effects, restoring SOD1 levels to control values. In contrast, thioredoxin reductase 1 (TrxR1) expression was significantly reduced following Rot exposure, either with or without LM11A-31, as assessed by WB analysis. No significant changes were observed in the protein levels of SOD2 (Figure 5G) or Catalase (Figure 5H) across the three groups investigated.

**FIGURE 6:**
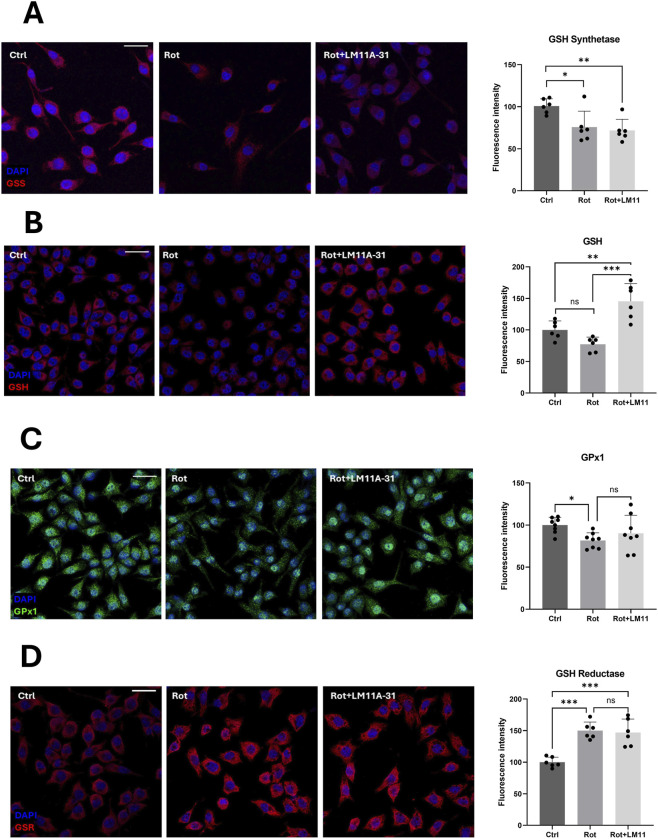
LM11A-31 modulates glutathione-dependent redox processes in microglia. **(A)** Representative confocal microscopy images (left panel) and quantitative fluorescence intensity analysis (right panel) of glutathione synthetase (GSS) staining (red) in BV2 cells treated with vehicle (Ctrl), 50 nM rotenone (Rot), or co-treated with LM11A-31 (Rot+LM11A-31) for 24 h. DAPI (blue) was used for nuclear counterstaining. N = 6. Magnification: ×40. Scale bar = 25 μm. **(B)** Representative confocal microscopy images (left panel) and quantitative fluorescence intensity analysis (right panel) of reduced glutathione (GSH) staining (red) in BV2 cells following the indicated treatments. DAPI (blue) was used for nuclear staining. N = 6. Magnification: ×40. Scale bar = 25 μm. **(C)** Representative confocal microscopy images (left panel) and quantitative fluorescence intensity analysis (right panel) of GPx1 staining (green) in BV2 cells. DAPI (blue) was used for nuclear counterstaining. N = 8. Magnification: ×40. Scale bar = 25 μm. **(D)** Representative confocal microscopy images (left panel) and quantitative fluorescence intensity analysis (right panel) of glutathione reductase (GSR) staining (red) in BV2 cells treated as described above. DAPI (blue) was used for nuclear counterstaining. N = 6. Magnification: ×40. Scale bar = 25 μm. Data are presented as mean ± SD. Dots represent individual biological replicates. Statistical analysis was performed using one-way ANOVA followed by Tukey’s *post hoc* test. *p < 0.05; **p < 0.01; ***p < 0.001.

Given glutathione (GSH) primary role as the main non-enzymatic antioxidant in cellular redox homeostasis, we investigated the impact of Rot exposure on GSH-related system (Figure 6). GSH immunoreactivity was used as an indicator of protein S-glutathionylation, i.e., a reversible post-translational modification protecting proteins from irreversible oxidative damage (Kalinina and Novichkova, 2023). Although Rot treatment induced a decreasing trend in S-glutathionylation levels displayed, this effect did not reach statistical significance, whereas p75NTR modulation by LM11A-31 significantly increased GSH immunoreactivity compared to both control and rotenone-treated cells (Figure 6B). In parallel, Rot exposure significantly reduced glutathione peroxidase 1 (GPx1) signal (Figure 6C), a key enzyme involved in glutathione-dependent hydrogen peroxide detoxification, while LM11A-31 treatment restored GPx1 expression to control values. Conversely, LM11A-31 did not significantly affect either glutathione synthase (GSS) or glutathione reductase (GSR) immunofluorescence levels, which were respectively decreased (Figure 6A) and increased (Figure 6D) following Rot exposure.

## 4 Discussion

Progressive dopaminergic degeneration in PD arises from interconnected mechanisms, including mitochondrial dysfunction, oxidative stress, and chronic neuroinflammation (Surmeier et al., 2017; Hirsch and Standaert, 2021; Ma et al., 2024). Dopamine replacement therapy with levodopa remains the gold standard for symptomatic management of PD, though failing to prevent the neurodegenerative process (Kalia and Lang, 2015; Aradi and Hauser, 2020). This therapeutic limitation highlights the urgent need for disease-modifying strategies to address upstream processes that contribute to neuronal vulnerability. Modulation of neurotrophin signaling via p75NTR, using the small molecule LM11A-31, has emerged as a promising approach (Xiong et al., 2022), although the mechanisms underlying its protective effects in PD are yet to be fully understood.

To shed light onto this issue, the present study addresses pharmacological modulation of p75NTR following Rot insult in BV2 microglial cells. We here demonstrate that LM11A-31 mitigates Rot-induced redox imbalance. Exposure to mild doses (50 nM) of Rot, to reproduce PD-like cellular alterations *in vitro*, resulted in a significant upregulation of p75NTR, indicating activation of stress-associated signaling cascades converging on this receptor. Such increase is consistent with previous reports describing its upregulation in pathological contexts (Colombo et al., 2012; Brito et al., 2013), supporting the notion that p75NTR participates in cellular responses to neurotoxic challenges.

Functionally, LM11A-31 improved cell survival and prevented apoptotic engagement, suggesting that p75NTR modulation counteracts pro-degenerative signaling triggered by Rot. Indeed, Rot administration reduced cell viability, activating apoptotic pathways, as assessed by TUNEL assay, a well-established method that detects DNA strand breaks generated by endonuclease activity during programmed cell death (Kyrylkova et al., 2012). Consistently, immunofluorescence analysis of cleaved caspase-3, a key regulatory protease in apoptosis (Hussar, 2022), revealed significantly increased signal intensity after Rot administration, suggesting the activation of the apoptotic pathway. Remarkably, LM11A-31 reduced the percentage of TUNEL-positive cells, and significantly decreased caspase-3 immunoreactivity, suggesting interference with upstream death signaling cascades. The concomitant decrease in TUNEL positivity and cleaved caspase-3 immunoreactivity suggests that LM11A-31 attenuates apoptotic pathway engagement in rotenone-treated BV2 cells. Noteworthy, confocal microscopic observation supports a predominantly cytoplasmic distribution of caspase-3. While its nuclear localization is classically associated with apoptotic execution, cytoplasmic active caspase-3 may suggest alternative roles of the protease, specific to microglial cells, consistent with previous studies. Indeed, Burguillos and coll. (2011) and subsequent work by Kavanagh and coll. (2014) have shown association of caspase-3-dependent signaling with microglial activation (Burguillos et al., 2011; Kavanagh et al., 2014). Therefore, the reduction of cytoplasmic cleaved caspase-3 observed after LM11A-31 treatment may reflect not only a decrease in apoptotic cell death, but even a dampening of caspase-3-related microglial activation.

Additionally, scanning electron microscopic (SEM) analysis confirmed Rot-triggered apoptotic changes. Typical morphological alterations, commonly associated with apoptosis (Elmore, 2007), such as surface irregularities, including membrane blebbing, ruffled plasma membrane, and decreased cell spreading, were invariantly observed in Rot-exposed cells (Zhang et al., 2018). It is worth mentioning that Rot administration has been associated with necroptosis, a form of regulated cell death, featuring morphological signs of necrosis, such as membrane permeabilization, together with the activation of death receptors (Roy et al., 2023). However, our TUNEL and ultrastructural results support a canonical apoptotic process, as plasma membranes appeared intact. This seeming discrepancy may be explained considering the different cellular models (neuroblastoma vs. microglial cell line) and the doses of the neurotoxin (100 µM vs. 50 nM) employed. On the other hand, the effects of Rot on actin network reported by the same group (2023), are in line with our data, showing overall cytoskeletal derangement leading to retraction of cell processes. Indeed, Rot-treated cells exhibited the disruption of the microtubule network, characterized by the loss of the tubulin filamentous architecture (Choi et al., 2011; Sokolowski et al., 2014).

In contrast to all the cellular abnormalities detected following Rot exposure, LM11A-31–cotreated cells showed regular features, closely similar to control. Smooth surfaced membranes, longer cell processes and the absence of apoptotic blebs or bodies could be ultrastructurally observed by SEM and were associated with preserved cytoskeleton, featuring radially organized tubulin filaments. The rescued morphology suggests that p75NTR modulation not only reduces molecular markers of apoptosis but also maintains structural stability at both intracellular and surface levels. Indeed LM11A-31 may exert a specific protective action toward cytoskeletal components, by both inhibiting their caspase-mediated proteolysis during apoptosis, and preventing their oxidative damage, thanks to reduced ROS production.

However, although the increase in TUNEL-positive cells indicates the occurrence of DNA fragmentation, the marked reduction in total cell number suggests that Rot-induced cytotoxicity may involve a broader spectrum of cellular damage. This finding points to a complex cell death scenario, in which DNA fragmentation represents only one component of the overall cell loss observed after Rot exposure. In this context, the marked increase in lipid peroxidation detected by BODIPY™ 581/591 C11 staining is particularly relevant, as it may indicate the contribution of oxidative membrane damage and possibly ferroptosis-related mechanisms. Nevertheless, this interpretation remains speculative, and further dedicated experiments will be required to directly establish the involvement of ferroptosis in this model. While neuronal vulnerability is an invariant feature of PD, the role of non-neuronal cells, particularly microglia, is yet to be defined, in terms of passive target or active player in disease progression. Increasing evidence indicates that indeed this cell population actively contributes to the propagation of neurodegeneration within the nigrostriatal system (Tansey and Goldberg, 2010; Halliday and Stevens, 2011). Due to their intrinsic functional plasticity, microglia can dynamically shift between pro- and anti-inflammatory states, thereby coordinating immune surveillance and neuronal support (Tang and Le, 2016; Guo et al., 2020; Augusto-Oliveira et al., 2025). Persistent redox imbalance can compromise this regulatory flexibility, leading to sustained microglial activation and to the amplification of inflammatory signaling (Subramaniam and Federoff, 2017).

Consistent with this framework, Rot exposure induced a pronounced activation profile in BV2 cells, as evidenced by the increased expression of Iba1 and CD68, two established markers of reactive microglia (Jurga et al., 2020). This molecular upregulation was accompanied by cell body enlargement, as shown by phalloidin staining, a morphological feature frequently related to an activated, pro-inflammatory phenotype (Davis et al., 2017). This change was also accompanied by enhanced intracellular immunoreactivity for TNF-α and IL-6, suggesting that Rot-induced microglial reactivity may be associated with a pro-inflammatory intracellular profile. Notably, LM11A-31 co-treatment abrogated the Rot-associated increase in TNF-α and IL-6 signals, bringing their levels closer to those observed under Ctrl conditions. Such anti-inflammatory action, supporting a transition of microglia to a more resilient phenotype, is consistent with previous reports, showing that pharmacologically targeting p75NTR blunts inflammation in various experimental models (Elshaer et al., 2019; Mirzahosseini and Ishrat, 2024).

Microglial activation are reportedly linked to oxidative stress, arising from an imbalance between free radicals and antioxidant defenses. NOXs represent the primary enzymatic sources of ROS and play a significant role in PD development (Belarbi et al., 2017). This notion derives from studies on MPTP mouse models and human tissues, showing elevation of NOX2 levels, which co-localize with CD68 in activated microglia, and concomitant phosphorylation of its regulatory subunit p47^PHOX^, demonstrating robust enzyme activation in the Substantia Nigra (Wu et al., 2003; Huh et al., 2011). Consistently, our data revealed that Rot exposure altered the expression of NOX-associated components, together with increased p47^PHOX^ immunoreactivity, suggesting enhanced activation of oxidative stress-related pathways. A concurrent increase in p22^PHOX^, the membrane-bound subunit essential for oxidase stability and proper regulation of enzyme activity, further supports the presence of an active enzymatic configuration. The induction of NOXs suggests that Rot amplifies ROS generation in microglia, potentially establishing a feed-forward loop between oxidative stress and inflammatory activation. Pharmacological modulation of p75NTR significantly reduced Rot-induced increase in NOXs expression, as well as in p47^PHOX^ and p22^PHOX^ subunits, indicating that LM11A-31 acts upstream of redox balance, limiting ROS production by suppressing oxidase assembly and stability. In parallel with NOX-related alterations, MitoSOX™ Red staining showed that Rot markedly increased superoxide production, highlighting the involvement of the mitochondrial compartment in Rot-induced oxidative stress. LM11A-31 significantly reduced MitoSOX™ Red fluorescence, although values remained above controls, suggesting a partial attenuation of mitochondrial ROS accumulation.

Several transcription factors coordinate the expression of genes involved in redox homeostasis and inflammatory signaling. In our model, Rot exposure showed the nuclear localization of PPARα and Nrf2, consistent with their activation, whereas LM11A-31 treatment significantly blunted this effect. The enhanced nuclear presence of these transcription factors likely reflects an adaptive response to counteract the redox disturbance induced by the toxic insult, in line with the well-established role of PPARs and Nrf2 in promoting the transcription of key antioxidants and cytoprotective enzymes, such as SODs, catalase, and Gpx1 (Takahashi et al., 2002; Billiet et al., 2008; Moreno and Cerù, 2015; Shin et al., 2016). Despite the evident nuclear translocation of redox-sensitive transcription factors, particularly Nrf2, the absence of a marked increase in downstream antioxidant enzymes may reflect a temporal dissociation between Nrf2 activation and the transcriptional upregulation of its target genes. Indeed, Nrf2 nuclear accumulation can precede, and not immediately translate into antioxidant enzyme induction, suggesting a delayed transcriptional response, consistent with previous reports (Zhang et al., 2017). Concerning PPARα, it is conceivable that, in these challenging conditions, its transcriptional activity mainly targets genes related to lipid/glucose metabolism to cope with energy imbalance (Kersten, 2014).

The glutathione system serves as a key intracellular redox buffer and is tightly regulated to maintain cellular homeostasis. Rot exposure disrupted this balance by directly impairing glutathione synthetase, aligning with earlier research (Huang et al., 2023), and reduced *de novo* GSH synthesis. Additionally, a significant increase in glutathione reductase levels was observed, which likely represents a compensatory mechanism to maintain GSH levels by enhancing the recycling of oxidized glutathione (GSSG). Notably, treatment with LM11A-31 did not significantly alter the levels of glutathione-metabolizing enzymes. In contrast, LM11A-31 treatment increased protein S-glutathionylation. While S-glutathionylation is often considered a marker of oxidative stress, it is also a reversible redox-dependent modification involved in the regulation of signaling pathways and protection of cysteine residues from irreversible oxidation (Xiong et al., 2011).

## 5 Conclusion

**FIGURE 7:**
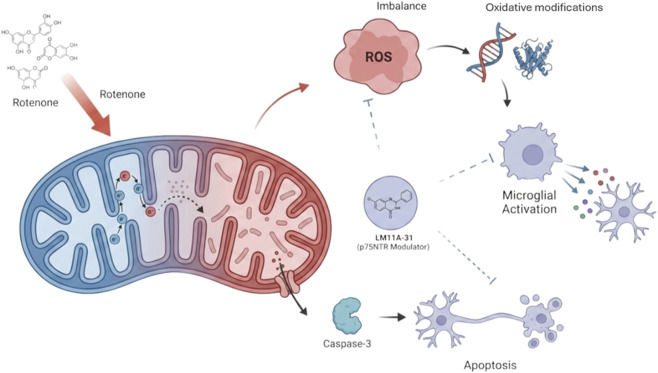
Mechanistic overview of rotenone-induced neurotoxicity and LM11A-31-mediated protection.

In conclusion, the present findings further support the central role of oxidative stress and neuroinflammation in driving microglial dysfunction associated with PD-related toxic insults. Our data provides strong evidence that pharmacological modulation of p75NTR via LM11A-31 effectively counteracts Rot-induced redox imbalance, apoptotic signaling, cytoskeletal derangement and pro-inflammatory activation in BV2 microglial cells (Figure 7). Targeting p75NTR may therefore represent a more integrated therapeutic strategy compared with approaches exclusively focused on antioxidant or anti-inflammatory mechanisms. Despite these promising results, several aspects require further investigation. PD is a multifactorial disorder in which neuronal–glial interactions, genetic susceptibility, and environmental factors converge to shape disease progression. Thus, validating the protective effects of LM11A-31 in more complex experimental systems, *e.g.,* neuronal cultures in microglia-conditioned medium, neuron–microglia co-cultures, and brain organoids*,* will be essential. Moreover, a deeper characterization of the p75NTR intracellular signaling cascades, will be crucial to clarify how its modulation reshapes redox-sensitive and inflammatory networks. A more detailed mechanistic understanding may ultimately facilitate the development of disease-modifying strategies aimed at slowing, rather than alleviating, the progression of Parkinsonian neurodegeneration.

## Data Availability

The raw data supporting the conclusions of this article will be made available by the authors, without undue reservation.
