# Supplementary material for: Pharmacological modulation of p75 neurotrophin receptor in microglial cells improves resilience to rotenone cytotoxicity
Source: Front Pharmacol. 2026 Jul 20;17:1895046. doi: 10.3389/fphar.2026.1895046 (PMC13429958; doi:10.3389/fphar.2026.1895046)
Supplement: Supplementary file 1 [file DataSheet1.pdf]

# *Supplementary Material*

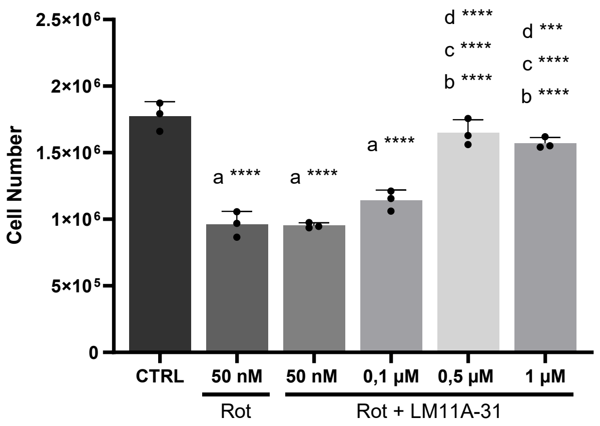


**Supplementary Figure 1.** Representative cell count quantification of BV2 cells treated with vehicle (Ctrl), 50 nM rotenone (Rot), or co-treated with LM11A-31 (0.05, 0.1, 0.5, or 1 µM). Data are expressed as mean ± SD, $N = 3$. Statistical analysis was performed using one-way ANOVA followed by Tukey's post hoc test. "a" indicates statistical significance vs. Ctrl; "b" indicates statistical significance vs. Rot group; "c" indicates statistical significance vs. Rot + LM11A-31 0.1 µM group;  d" indicates statistical significance vs. Rot + LM11A-31 0.5 µM group. $***p < 0.001$, $****p < 0.0001$.

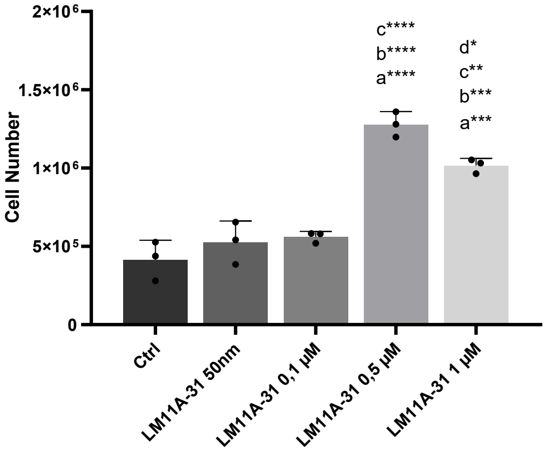


**Supplementary Figure 2.** Representative cell count quantification of BV2 cells treated with vehicle (Ctrl), and with LM11A-31 (0.05, 0.1, 0.5, or 1 µM) for 24 h. Data are expressed as mean ± SD, $N = 3$. Statistical analysis was performed using one-way ANOVA followed by Tukey's post hoc test. "a" vs. Ctrl group; "b" vs. LM11A-31 0.05 µM group; "c" vs. LM11A-31 0.1 µM group; "d" vs. LM11A-31 0.5 µM group; "e" vs. LM11A-31 1 µM group. ***p < 0.001, ****p < 0.0001.

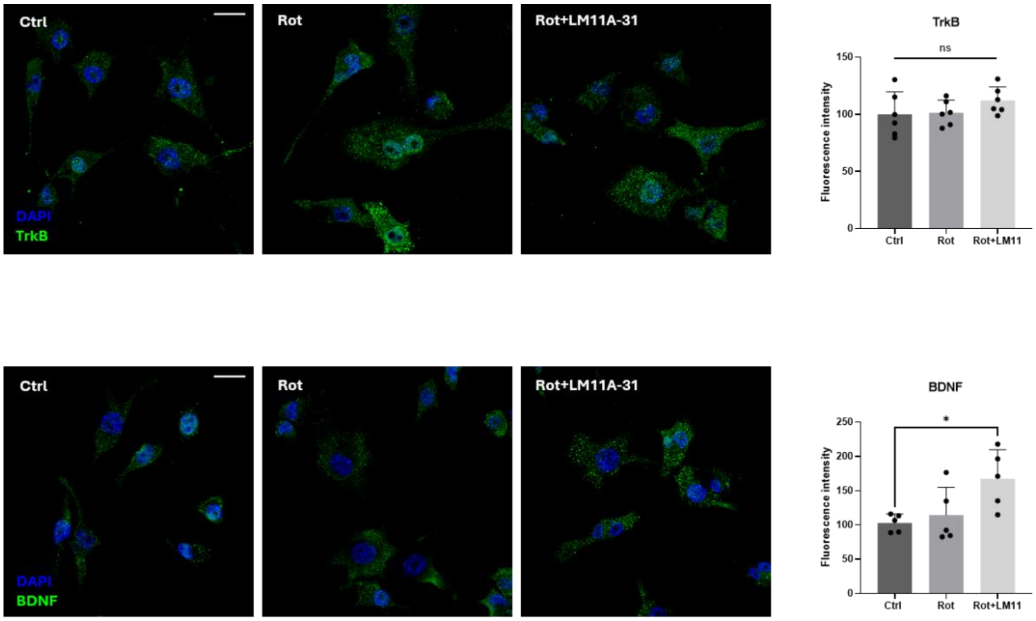


**Supplementary Figure 3**. Representative confocal microscopy images (left panel) and quantitative fluorescence intensity analysis (right panel) of TrkB and BDNF staining (Green) in BV2 cells treated with vehicle (Ctrl), 50 nM rotenone (Rot), or co-treated with LM11A-31 (LM11A-31) for 24 h. DAPI (blue) was used for nuclear counterstaining. N = 5-6. Magnification: 40×. Scale bar = 25µm
